# Investigation of mutational spectrum in cytochrome P4501B1 (CYP1B1) as the principal cause of primary congenital glaucoma

**DOI:** 10.12669/pjms.39.2.7081

**Published:** 2023

**Authors:** Tazeen Zahid, Muhammad Umer Khan, Aymn Zulfiqar, Fatima Jawad, Anosh Saleem, Ahmad Raza Khan

**Affiliations:** 1 Tazeen Zahid DMLS, MPhil Scholar., Institute of Molecular Biology and Biotechnology, The University of Lahore, Lahore, Pakistan; 2 Muhammad Umer Khan, MPhil, PhD., Institute of Molecular Biology and Biotechnology, The University of Lahore, Lahore, Pakistan; 3 Aymn Zulfiqar, DMLS., University Institute of Medical Laboratory Technology, Faculty of Allied Health Sciences, The University of Lahore, Lahore, Pakistan; 4 Fatima Jawad, DMLS., University Institute of Medical Laboratory Technology, Faculty of Allied Health Sciences, The University of Lahore, Lahore, Pakistan; 5 Anosh Saleem, DMLS., University Institute of Medical Laboratory Technology, Faculty of Allied Health Sciences, The University of Lahore, Lahore, Pakistan; 6 Ahmad Raza Khan, DMLS., University Institute of Medical Laboratory Technology, Faculty of Allied Health Sciences, The University of Lahore, Lahore, Pakistan

**Keywords:** Primary congenital glaucoma, Intraocular pressure, Cytochrome P450, Mutation, Consanguinity

## Abstract

**Objective:**

To identify the genetic variants in the *CYP1B1* gene associated with Primary Congenital Glaucoma (PCG) and to predict its pathological effect.

**Method:**

A descriptive study was conducted in the time period of nine months (September 2021-May 2022) after the ethical approval was taken from The Children Hospital and Institute of Child Health (CH & ICH). Two milliliters of the blood sample from PCG-affected individuals were collected in EDTA vacutainers and genomic DNA was extracted by a phenol-chloroform method. The semi-quantification of extracted DNA was done by agarose gel electrophoresis. PCR amplification was performed by specific primers of *CYP1B1* gene then termination sequencing (di-deoxy) was done to detect the genetic variants. Different bioinformatics tools such as BLAST, Ensembl, Clustal Omega, Polyphen and SIFT were used for the further analysis of mutation causing the disease.

**Result:**

A total of 85% of patients were bilaterally affected, while 15% were unilaterally affected. Mutation analysis identified five non related known variants. Two missense mutations (c.355 G/T p.A119S and c.685G/A p.E229K) occurred in 94% patients and intragenic SNP occurred in 29% patients along with the 1% somatic (c.693C/A p.F231L) and stop gained mutation (c.840C/A p.C280*).

**Conclusion:**

Genetic analysis in the current study showed that 85% of PCG affected patients were due to the *CYP1B1* mutation, and disease heterogeneity might be reduced through genetic counseling.

## INTRODUCTION

Primary congenital glaucoma (PCG), a visual debilitate abnormality, is a non-syndromic, idiopathic, intrinsic developmental impairment of the anterior chamber angle and abnormal cupping of the optic nerve head manifesting in the third trimester of gestation.^1^ This developmental detention results in photophobia, hyper lacrimation, and blepharospasm. The early existence of PCG may probably be identified within the initial three years of birth.^2^ National analysis of blindness and ocular impairment disclosed that glaucoma is the fourth major source of blindness in the Pakistani populace (7.1%). The frequency of PCG is not completely known in Pakistan.^3^,^4^ In China, 1:24,941 populace and in Australia 1:30,000 individuals are afflicted by PCG.^5^,^6^

The prevalence of PCG in western communities ranges between 1:10,000 and 1:70,000.^7^ An incidence rate of 1:2766 has been reported in the extremely consanguineous population like in Saudi Arabia, as it is exceptionally higher in Southern India in addition to Slovakia, its frequency fluctuates somewhere in the extent of 1:1250 and 1:3300, respectively.^8^ Increased pervasiveness of PCG has been observed in the consanguineous relations. The incidence of PCG is typically higher in male as compared to female.^9^ The exact etiology of PCG is not fully acclaimed.

A significant extent of heterogeneity has been detected in patients with PCG possessing disease-associated loci, pervasiveness of abnormality, and penetrance insufficiency amid different populaces.^10^ So far, four distinct hereditary loci were identified to be associated with PCG. Even though particular genealogical etiology and origin have yet required to be confirmed, several significant genes, *CYP1B1*, Angiopoietin-1 receptor tyrosine endothelial kinase *(ANGPT1)*, Latent transforming growth factor-beta binding protein-2 *(LTBP2*), are involved in the pathogenesis of PCG.^11^ Mutation variations in cytochrome P450 (*CYP1B1*) gene is a frequent genetic predisposition linked with primary congenital glaucoma and is specifically engaged in forming the trabecular meshwork in eye.^2^ Although more than 150 *CYP1B1* mutations have been discovered in PCG reported patients globally, they just exhibit 87% occurrence in hereditary population and 25-27% of cases in heterogeneous ethnicities.^12^

The ratio of *CYP1B1* mutations differ considerably amongst various populace. About 14.9% PCG cases in the US and 20% Japanese cases have been spotted with pathogenic *CYP1B1* alleles. Similarly, ~15.2%, 75.9%, and 47.7% patients are reported with *CYP1B1* mutated alleles in China, Saudi Arabia, and Morocco, respectively.^13^ Numerous studies have detected the *CYP1B1* variants that are responsible for causing primary congenital glaucoma among Pakistani populace.^14^,^15^ In the Pakistani population five already known variants and three novel (c.1436A>G, c.542T>A, and c.1325delC) variants of CYP1B1 were spotted.^16^ Mutational analysis of CYP1B1 gene among 38 Pakistani families affected with primary congenital glaucoma revealed the presence of four known mutations (p.G61E, p.R368H, p.R355X and p.R390H) and a novel mutation in intron 2.^17^ Nevertheless, mutations of CYP1B1 gene have been narrated generally in 37.7% of the involving Pakistani lineages in cooperated genetic analysis.^18^ Approximately 1.8 million glaucoma cases have been reported in Pakistani population and about half of them have thus far lost their vision permanently as a result lack of awareness about the diagnostic procedures and treatment.^19^ Evaluation analysis for PCG comprise the assessment of intraocular pressure (IOP) at an initial phase of sedation, pivotal length measurement (mm), corneal distance (mm) level, biomicroscopic estimation of anterior segment, gonioscopy, fundoscopy and calculation of cup-disc ratio. The effectual prognostic approaches for PCG include trabeculectomy and goniotomy; preference relies on the severity.^20^

To avert the advancement of PCG, an increased awareness has been developed regarding genetic counseling. The hereditary pattern of the disease can be defined by the formation of a familiar lineage.^11^ Thus, diverse measures have to be acquired to minimize PCG develop into the most preeminent aspect for irreversible ocular abnormality. Based on the previous investigations, current study was designed to determine the genetic variants associated with PCG in Pakistani population and to deliver a detailed information about *CYP1B1* gene related to PCG.

## METHOD

### Study subjects and clinical assessment:

A descriptive study was carried out in The Children Hospital and Institute of Child Health (CH&ICH) and University Institute of Medical Lab Technology, The University of Lahore, Pakistan after the approval by the institutional ethical committee (REC-UOL-/36-09/2022). The study was conducted in the time period of nine months (September 2021-May 2022). Ethical principles for medical research involving human subjects as adopted by the World Medical Association Declaration of Helsinki (DoH/Oct2008) were taken into consideration. A total of 20 blood samples were collected after the written informed consent of the patient’s guardians. Blood samples of only clinically diagnosed PCG patients were collected in EDTA vial. Based on the clinical diagnosis, patients fulfilling the criteria of having age of onset up to three years, IOP greater than 21 mmHg without any treatment and having cup disc ratio greater than 0.3 were included in the study, while those reported with other ocular abnormality beside PCG were excluded from this study.

### Mutation analysis:

The extraction of genomic DNA was carried out by using in housed developed phenol-chloroform method. Quantification was checked on 1% Agarose Gel. The specific genomic DNA sequence was amplified by using two sets of primer pairs with a sequence 5→3` (Forward primer GGCCATTTCTCCAGAGAGTC) and (Reverse primer AACTCTTCGTTGTGGCTGAG). Ensemble Genome Browser 108 (GRCh38.p13) was used to retrieve *CYP1B1* genomic sequence. By using Primer3 genome Browser, specific primers were designed and were required to get manufactured commercially. Optimization of primer was performed by using gradient PCR. A polymerase chain reaction reaction was performed using 25µl reaction volume by adding PCR Master Mix (2X; Thermo scientific, #Ko171 and Lot# 00186508).

The components required to prepare 25µl reaction mixture contained 9.7µl of nuclease free water, 12.5µl of 2X PCR master mix, 0.4μl for both forward and reverse primers, 2.0μl of genomic DNA. A three step PCR reaction was performed involving initial denaturation at 95°C for five minutes, the next step involved further 35 cycles of denaturation, annealing and extension at 95°C for one minute, 59.5°C for 30 sec and 72°C for 30 second, respectively. Final extension was carried out at 72°C for 10 minutes. Detection of amplified PCR product of 829bp was verified on 1% agarose gel electrophoresis. PCR product was purified by ethanol precipitation method and then the clinically diagnosed PCG samples were sent for sequencing.

### In Silico Analysis:

BLAST-2, Ensemble, Clustal omega, SIFT and Polyphen-2 were employed for in silico analysis. Chromas was used to check the chromatograms and obtained FASTA sequences for further in silico analysis. The BLAST was used to figure out the regions that were not similar with the wild type nucleotide sequences to determine the mutation. Ensemble was used for the interpretation of resultant variants and its effect on the related protein. PolyPhen-2 was employed to code nonsynonymous annotations of SNPs. Both polyphen-2 and SIFT were used to find out the impact of amino acid substitution on the function and structure of protein. While clustal omega was applied to generate multiple sequence alignments between three or more protein sequences of different species.

### Statistical Analysis:

Data was analyzed using IBM SPSS-27 IBM SPSS Statistics 28.0.1.1 (SPSS IBM Corporations USA).

## RESULTS

The results showed that 80% sampled population was male and the rest was female. Patients (85%) were found to be affected with PCG due to the mutation in *CYP1B1* gene. Three patients were unilaterally affected, while all other seventeen had bilateral ocular abnormality.

Mutation analysis revealed the presence of five different variants: Two missense mutations (c.685G/A p.E229K and c.355 G>T p.A119S), one somatic mutation (c.693C/A p.F231L), one stop gained mutation (c.840C/A p.C280*) and an intragenic SNP, c.142 C>G p.R48G. Missense mutation being the most common occurred in 94% patients, along with the somatic and stop gained mutation that appeared in the rest of the patients. After performing mutational analysis, a somatic mutation c.693C/A p.F231L was detected in heterozygous state. Mutation occurred by the codon change from TTC>TTA as a result of which the nucleotide sequence at 231 position that code for phenylalanine now code for leucine. c.685G/A p.E229K has been detected in two (11.7%) cases as a homozygous variant in one and a compound herterozygous variant in other.

**Fig.1:**
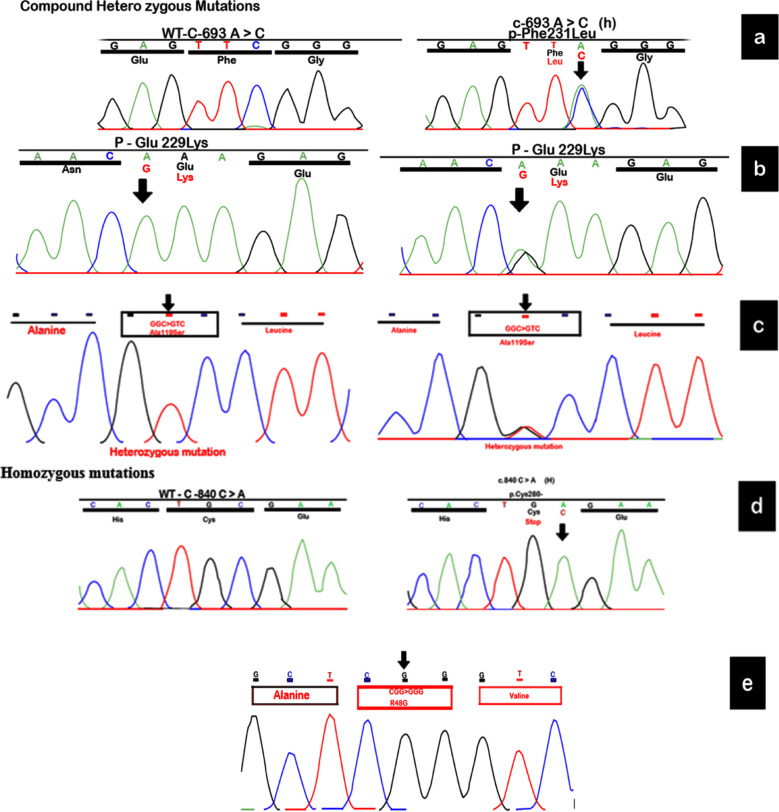
(a) Chromatogram showing the variation from TTC>TTA (p.F231L) (b) Homozygous and compound heterozygous mutation in p.E229K (c) c.355G>T p.A119S mutation resulting in homozygous and heterozygous variants (d) Nucleotide variation from TGC>TGA in p.C280* (e) Nucleotide variation from CGG>GGG (c.142p.R48G).

Similarly, a homozygous as well as heterozygous mutation c.355 G>T p.A119S has been identified in 76% patients, the highest ratio among all others mutations observed in this study. Homozygous mutation was observed in 38% patients while the rest of 61% patients revealed the mutation in heterozygous state. A stop gained variant c.840C/A p.C280* in homozygous state was detected in one patient. The resultant sequence TGC that code for cysteine now functions as a stop codon (TGA). An intragenic SNP, R48G, causing missense mutation has been identified in homozygous state in *CYP1B1* gene. Affected individuals have been found 29% to be with R48G (Fig.1).

**Fig.2:**
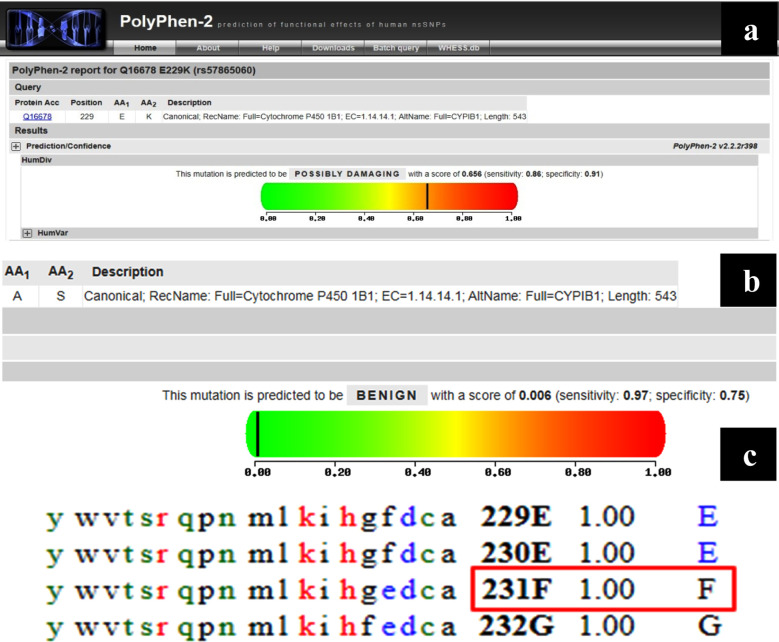
(a) Polyphen2 identifying the mutation as probably damaging (b) p.A119S and p.R48G were reported to be benign mutation (c) SIFT score is 1.00 in F231L mutation.

Variation in the nucleotide sequence was further confirmed by running it on BLAST. All the five detected mutations were analyzed by using Polyphen-2 to determine whether the variation had deleterious effect on function and structure of protein or not. As the score closer to one were predicted to have deleterious effect so Polyphen-2 described F231L and E229K mutations as probably damaging while A119S and p.R48G were reported to be benign with the score of 0.0006. Mutational variations were further analyzed by using SIFT to determine the impact of substitution of amino acid on the function of protein. SIFT score of 1.00 was obtained as a result of F231L and SNP p.R48G mutation thus indicating that the substitution can be tolerated without having any pernicious effect on the function of protein as only the mutations that scored between 0.0 to 0.05 would have deleterious effects (Fig.2).

Multiple sequence alignment of CYP1B1 proteins were performed by using Clustal Omega and Clustal w. CYP1B1 proteins exhibits the conservation of its residues at 231 and 229 positions among five different species. Thus, a missense variant (p.E229K) and a somatic variant (p.F231) affected amino acid residues were highly conserved in CYP1B1 protein orthologs. It was also observed that the sequence that codes for alanine at 119 positions in Homo sapiens, code for proline and serine in the other species. Similarly, cysteine at 280 position was conserved in all these species with the expectation in one specie that is Balaenoptera acutorostrata scammoni and another residue that code for arginine at 48 positions in homo sapiens, code for glycine in all other species, thus showing that amino acid residues were not conserved at position 119, 280 and 48 among these species (Fig.3). Three-dimensional structural analysis of CYP1B1 proteins was done by using Ensembl to further evaluate the possible effects of detected mutational variants on the three-dimensional structure of the encoded protein (Fig.4).

**Fig.3(a):**
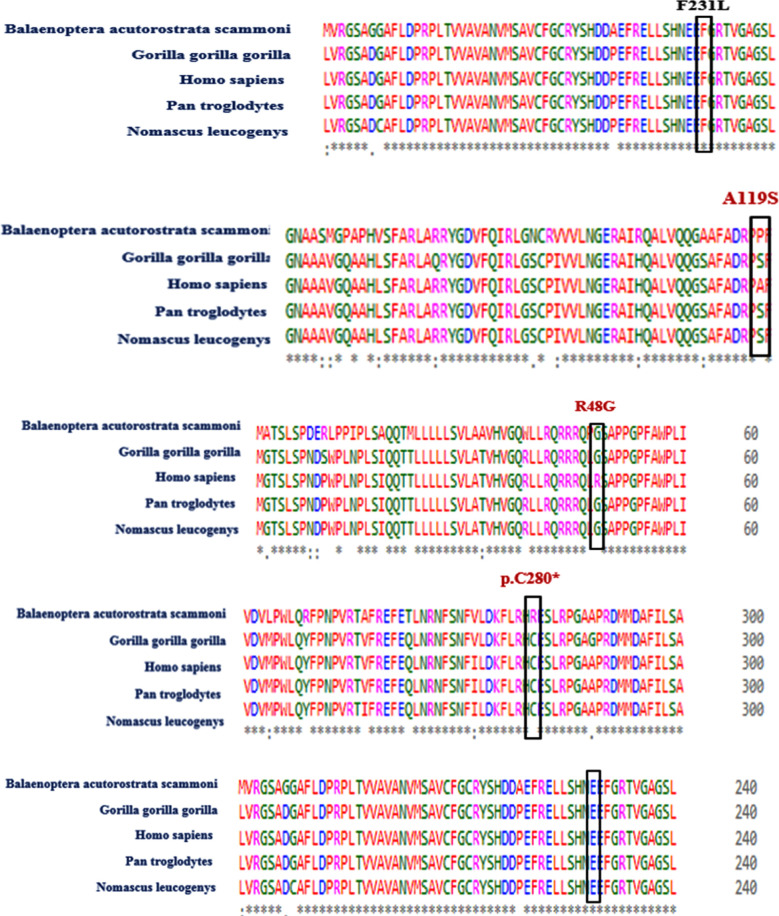
(a): Multiple sequence alignment of CYP1B1 proteins of affected individual with four different species.

**Fig.3(b):**
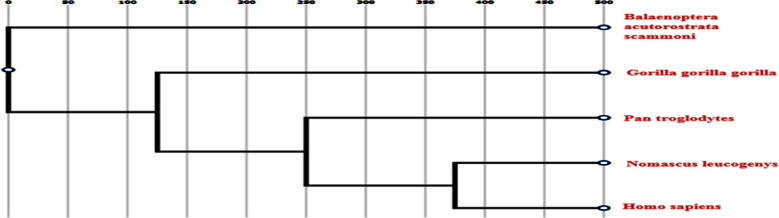
Phylogenetic tree obtained from Clustal w depicts the visual representation of the connection between these species. Nomascus leucogenys being closet to Homo sapiens, reflecting the maximum ancestral relationship with them.

**Fig.4:**
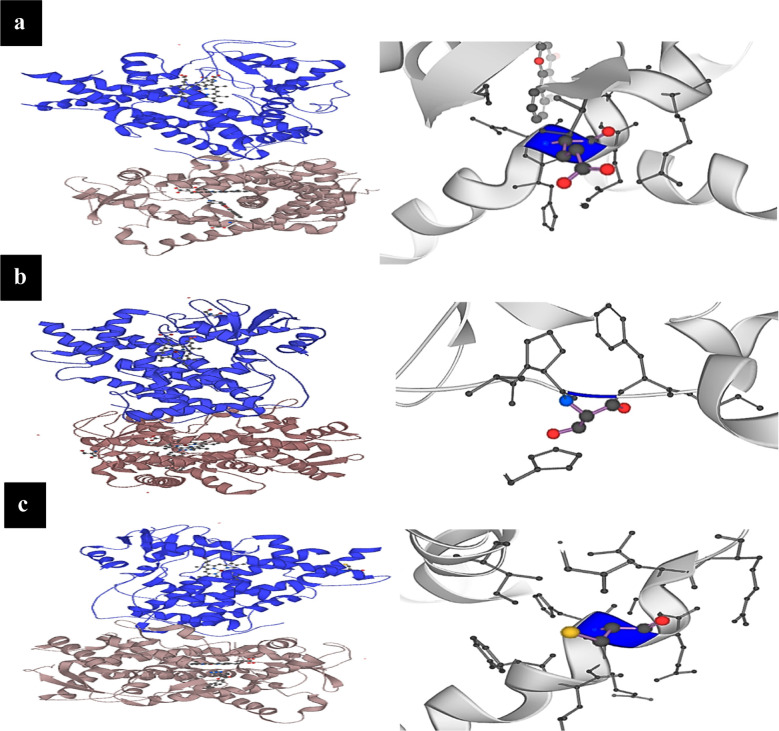
(a): 3D protein model of p.E229K. (b) 3D model of A119S. (c) 3D model of p.C280*.

## DISCUSSION

Primary congenital glaucoma is a rare condition which occurs primarily in neonates and can be a leading cause of blindness in children.^12^ Most of the cases of PCG come across with diverse variations in *CYP1B1* gene. The current findings showed that the individuals are affected with PCG in *CYP1B1*gene mutations separated with a phenotype of the disease. All the patients that turned out to be affected with primary congenital glaucoma were showing various pattern of mutations. 85 % of the patients were affected by the mutation on exon two, while the mutational cause in the rest of the individuals were not known yet.

The global reported data of variation indicates that mostly variations are heterogeneous with approximately ~70 alterations. According to another study which was also conducted in Lahore Pakistan, manifested that 20% of the PCG patients have the variant of *CYP1B*1 gene c.1169 G>A which leads to p.R390H on exon three. Furthermore it was reported that PCG patients revealed various mutations which includes, synonymous variant c.1347T>C present in 18 of the patients, 2 missense variants c.1358A>G in four patients while c. 1294G>C in two patients.^21^ *CYP1B1* mutations were seen in cohorts in a higher incidence from Turkey, Saudi Arabia (75%), Morocco (48%)^13^, Brazil (44%), along with India (44%).^22^

The present analysis revealed that missense mutation was the most spotted and common mutation in Pakistan, found in almost 88% patients while the rest of mutations were somatic mutation along with stop gain mutation and intragenic SNP. Five different variants found by mutational analysis were F231L, C280*, E229K, A119S and R48G. The study also revealed that all the variants of *CYP1B1* gene has an elevated level of homozygous A/A as well as heterozygous G/A mutations in males as compare to females. The mutations were detected by the sequencing of exon two; with exon boundaries along with protein coding region of *CYP1B1* gene using genomic DNA from affected patients of PCG.

The disease-causing mutation predicted by mutation tester c.693C/A p.F231L is a somatic mutation found heterozygously only in one patient. It was previously reported in Tunisia, in a homozygous pattern. Another mutation A119S was discovered as missense mutation and was expected to be benign.^23^ A119S mutation was also seen in a study conducted on thirty Brazilian non related PCG patients.^24^ A119S, a missense mutation occurred in thirteen patients, four were affected homozygously and the rest were heterozygous. A homozygous as well as compound heterozygous variant c.685G/A p.E229K detected in exon two at position 229 has been previously identified in Pakistani families affected with PCG. Previous studies discovered that E229K has no effect on an enzyme of *CYP1B1* gene. Various other studies reported that E229K was heterozygously identified in the healthy carriers and affected patients as well.^25^

Another study showed that homozygous along with compound heterozygous E229K allelic variant is closely linked with severe phenotype.^18^ A study conducted on fifty PCG patients along with fifty healthy individuals which were taken as a control. Out of fifty patients, five patients exhibited E229K mutation in heterozygous state.^26^ E229k mutation was also found in a study conducted in Iraq. 9% PCG patient’s revealed E229K mutation in heterozygous state as reported in previous studies. It was also observed in 7% healthy controls.^27^ The *CYP1B1* gene variation Arg48Glyc depict in this study that C>G (R48G) is a missense mutation and is not associated with disease.

Stoilov IR revealed that this R48G variant prevalence was 0.29% in the normal populations in Turkey along with UK. It was also reported that R48G gene variation prevalence was 0.295 in patients who were affected with PCG.^28^ The variant c.840C/A p.C280* a stop gain mutation detected in a homozygous pattern by ensemble which was previously reported in Japanese families heterozygously. It is one of the rarely known mutations causing PCG which shows a specific mutational spectrum.^29^ Thus based on the recent findings, it was concluded that the mutations in *CYP1B1* gene are the most predominant cause of PCG.

### Limitations:

Due to the financial limitations, the sample size was restricted. We also needed to restrict ourselves to sanger sequencing due to the financial burden.

## CONCLUSION

Primary congenital glaucoma is a complicated ocular defacement correlated with remarkable clinical and congenital heterogeneity. Mutational analysis revealed the presence of five different variants revealing two missense mutations (A119S and E229K) along with the somatic (F231L), stop gained (C280*) and intragenic SNP(R48G). Rapid and precise prognosis of PCG is significant, so that decisive measures can be instigated before irreversible impairment to the visual assessment. Further genetic counseling could play an important role in reduction of disease burden.

### Author Contributions:

**TZ, AZ and FJ** collected the blood samples and performed the experimental work.

**AS and ARK** wrote the initial manuscript.

**TZ and MUK** performed the in silico analysis.

**TZ and MUK** finalized the manuscript.

**MUK** supervised the whole project and is responsible for any future correspondence.
